# The clinical and pathological characteristics of lipid-rich carcinoma of the breast: an analysis of 98 published patients

**DOI:** 10.1186/s12905-023-02449-2

**Published:** 2023-06-08

**Authors:** Mengdi Zhang, Dongqing Pu, Guangxi Shi, Jingwei Li

**Affiliations:** 1 Shandong University of Traditional Chinese Medicine, No. 16369 Jingshi Road, Lixia District, Jinan City, 250014 Shandong Province China; 2 Department of Thyroid and Breast Diagnosis and Treatment Center, Affiliated Hospital of Shandong, University of Traditional Chinese Medicine, No. 16369 Jingshi Road, Lixia District, Jinan City, 250014 Shandong Province China

**Keywords:** Lipid-rich carcinoma, Breast cancer, Epidemiology, Pathology, Clinical features

## Abstract

**Background:**

Due to the small number of cases and few literature reports, the clinical treatment and prognosis of lipid-rich carcinoma of the breast are not summarized, which will lead to misdiagnosis and mistreatment and delay the patient’s condition. This study collected published case reports and analyzed the clinical characteristics of lipid-rich carcinoma of the breast in order to provide reference for early diagnosis and treatment of the disease.

**Methods:**

We performed a search using the PubMed, ClinicalTrials.gov, Embase, Cochrane Library, and China National Knowledge Infrastructure (CNKI) databases for publicly published case reports of lipid-rich carcinoma of the breast and obtained basic information of the patients such as country, age, sex, onset site, surgical method, pathology, postoperative treatment, follow-up time, and outcome (Table 9). The data were analyzed using Statistical Product Service Solutions (SPSS).

**Results:**

The mean age of the patients at diagnosis was 52.79 years and the median age was 53 years. Breast masses were the main clinical manifestations, with the upper outer quadrant (53.42%) being the most common. The treatment for lipid-rich carcinoma of the breast is mainly surgery plus postoperative adjuvant radiotherapy and chemotherapy. According to the results of this study, the surgical method recommended modified radical mastectomy (46.59%). Lymph node metastasis was found in 50.60% of the patients at the time of the first diagnosis. Patients who received postoperative adjuvant chemotherapy and radiotherapy had the highest disease-free survival and overall survival.

**Conclusion:**

Lipid-rich carcinoma of the breast has a short course of disease and early lymphatic or blood metastasis, and its prognosis is poor. In this study, we summarize the clinical and pathological characteristics to provide ideas for the early diagnosis and treatment of lipid-rich carcinoma of the breast.

## Introduction

Lipid-rich carcinoma of the breast (LRCB) is a very rare malignant tumor of the breast, accounting for 1–1.7% of all malignant breast tumors. LRCB is known to occur because tumor cells are rich lipids. Aboumrad was first discovered in 1963 [1]. In 1974, Ramos and Taylor officially named it lipid-rich carcinoma of the breast [2]. Later, clinical reports of LRCB gradually appeared, and it was determined to be an independent type of breast cancer by the new WHO classification in 2003. To date, fewer than 300 cases have been reported.

LRCB is a rare special type of breast cancer, which has attracted the attention of clinicians because of its high malignancy, rapid progress, poor prognosis and lack of specificity in clinical manifestations. Due to the small number of cases of LRCB, there is not much summary of clinical treatment and prognosis of this disease at present. The management of LRCB remains controversial because it is prone to malignant transformation. Most authors advocate surgical treatment in advance when surgical treatment can be performed once the diagnosis is confirmed or neoadjuvant chemotherapy followed by surgical treatment followed by postoperative adjuvant treatment. The purpose of the current study was to collect sufficient patient queues to study the epidemiological, clinical, and pathological features of LRCB in order to better understand this rare disease.

## Materials and methods

### Data sources and study patients

**Fig. 1:**
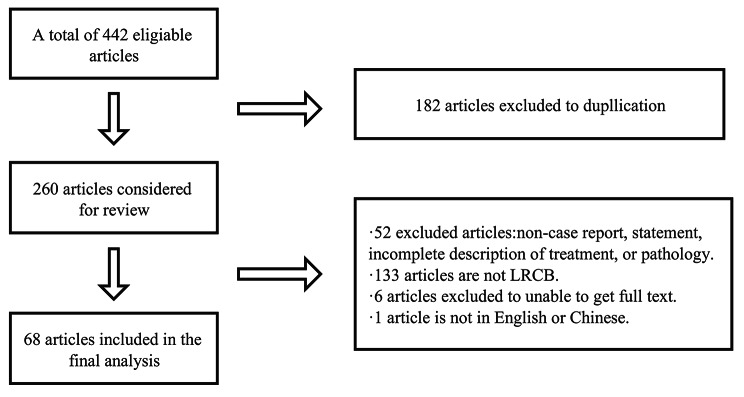
Literature review inclusion process

We performed a search using the PubMed, ClinicalTrials.gov, Embase, Cochrane Library, and CNKI databases from the date of their inception to November 30, 2021, without language restrictions, using the following search terms: “lipid-rich carcinoma of breast,” OR “lipid-secreting carcinoma of breast,” OR “lipid-rich breast cancer,” OR “lipid-secreting breast cancer”. All enrolled studies were confirmed based on the following criteria: (I) a clear diagnosis process is presented in the article, and (II) the third edition of the International Classification of Oncology Diseases (ICD-O-3.2) was used as a histological reference for diagnosis. The flow chart (Fig. 1) shows the identification of the LRCB and the reasons for its exclusion.

The following clinical and laboratory variables were studied:(I) country; (II) sex; (III) age at diagnosis; (IV) type of cancer; (V) whether the patients received chemotherapy and/or radiotherapy; (VI) whether and where the tumors metastasized; (VII) misdiagnoses (misdiagnosis criteria are obtained from the included literature); and (VIII) final results.

This is a retrospective study, and all the data are from published and available database documents. This study was exempted from ethical approval procedures, because no clinical trials or randomized controlled trials were conducted, and individual consent was waived.

### Statistical analyses

The demographic, clinical, and pathological characteristics of the patients were described utilizing simple summary statistics; the categorical and continuous variables were analyzed using chi-square analysis and Student’s t-test.

Statistical analyses were performed using the SPSS software (version 26.0; SPSS Inc., Chicago, IL, USA), and one-sample K-S tests were used to determine whether the data conformed to a normal distribution. All tests were two-sided, and a p-value < 0.05 was considered statistically significant.

## Results

### Epidemiological characteristics

**Fig. 2:**
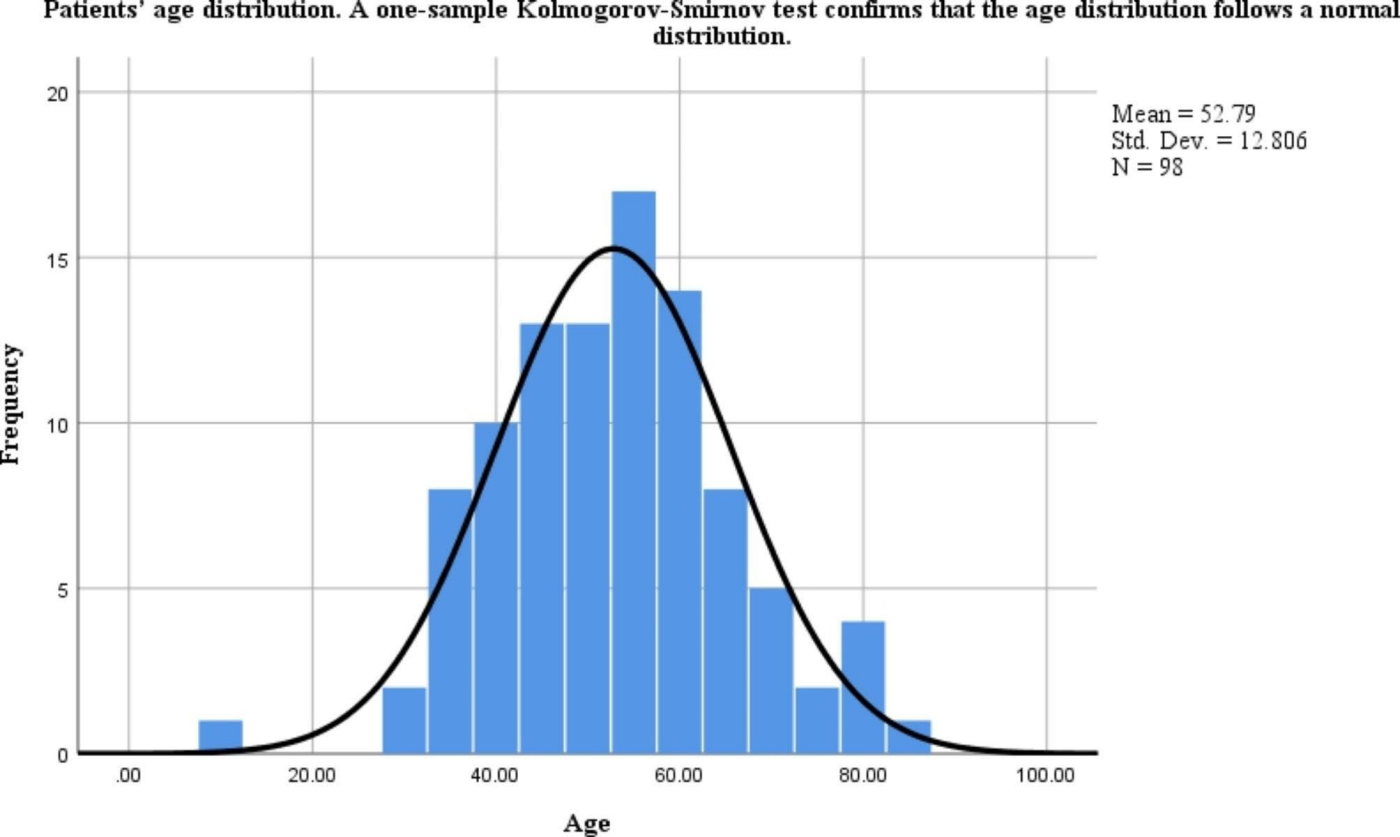
Patients’ age distribution. A one-sample Kolmogorov-Smirnov test confirms that the age distribution follows a normal distribution

**Fig. 3:**
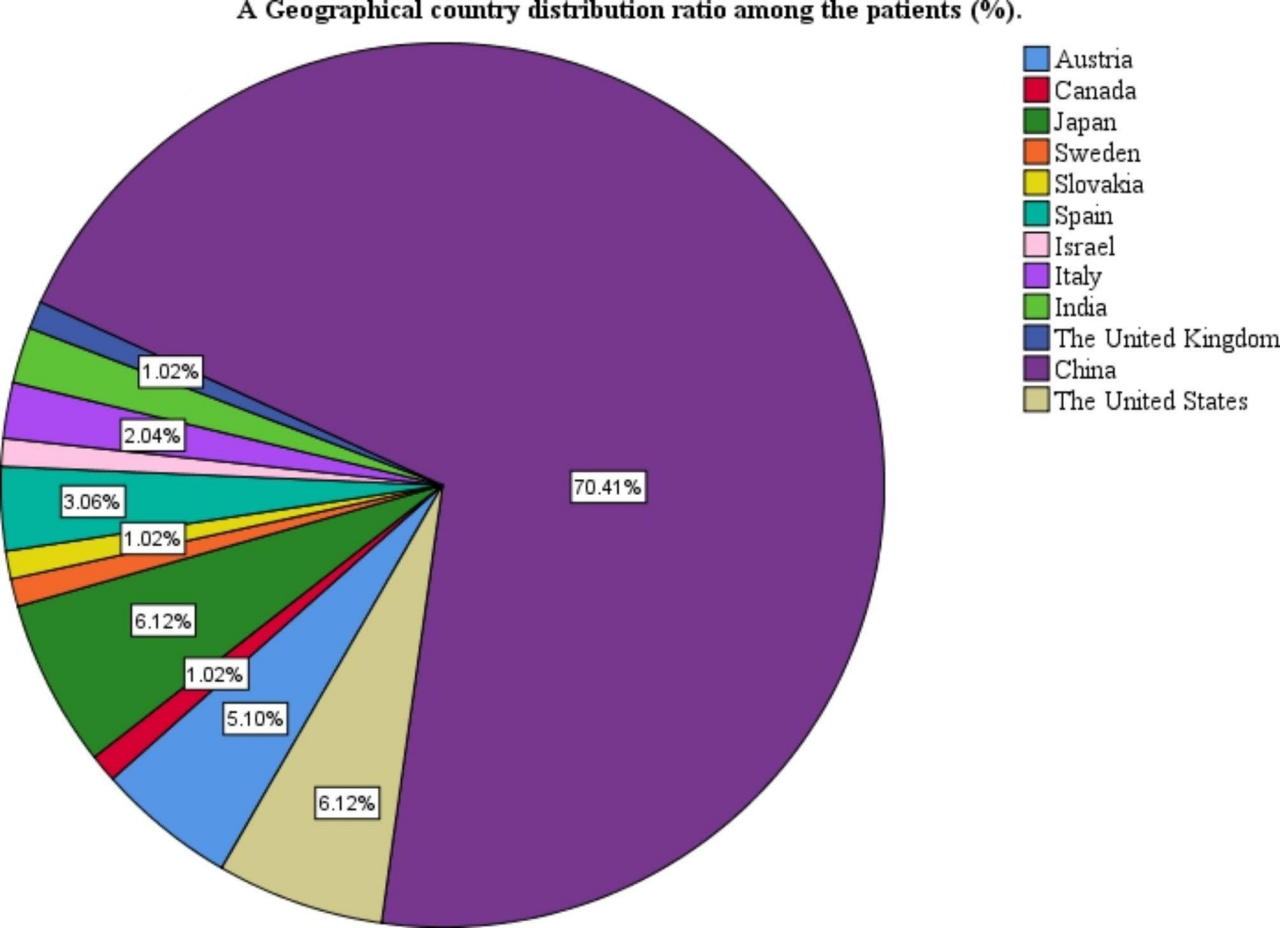
Geographical country distribution ratio among the patients (%)

**Fig. 4:**
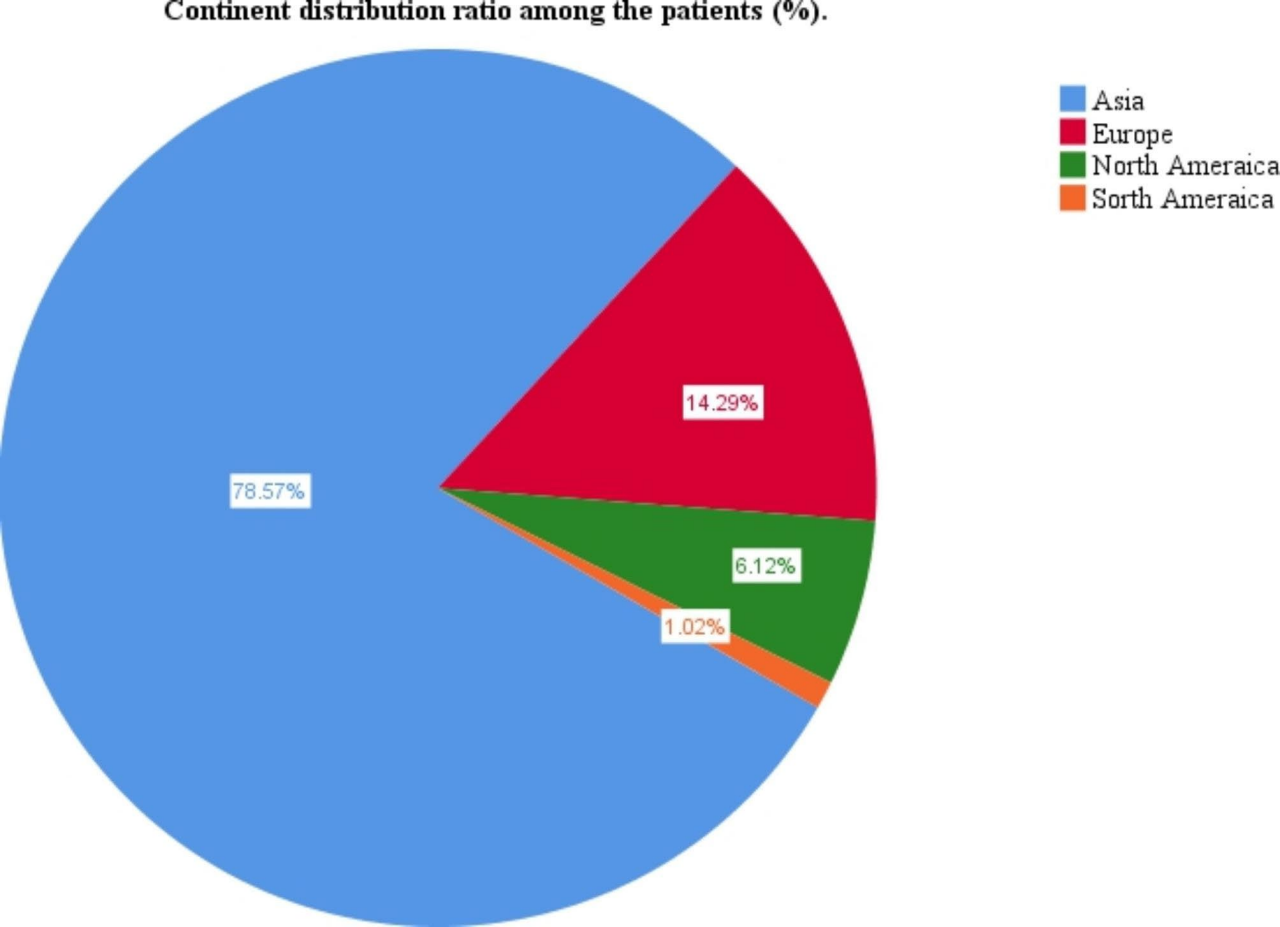
Continent distribution ratio among the patients (%)

This study included 68 articles (Fig. 1), involving 98 eligible patients, from 1963 to 2021. The patients were from 12 countries and four continents (Fig. 3), including China (69 cases, 70.41%), Japan (6 cases, 6.12%), and the United States (6 cases, 6.12%), which accounted for the top three countries (Fig. 3). Asia accounted for the largest proportion of the cases (78.57%) (Fig. 4). Among the 98 patients with available data regarding age, the average age at diagnosis was 52.79 years (Table 1) and the median age was 53 years (range, 10–83 years). The age groups with the highest incidence were 41–45 and 51–55 years. We found that the age at which the patient was diagnosed had a normal distribution (Fig. 2). In the 62 cases with definite follow-up times, the follow-up time ranged from 3 weeks to 20 years, with an average of 26.52 months (Table 1). Of the 64 patients who provided definitive follow-up results, 21 died (mortality rate: 32.81%).

**Table 1:** Summary of the clinical features lipid-rich carcinoma of the breast

Subjets	No.of patients	Mean ± SD
Age(year)	98	52.79 ± 12.81
Age(live)	38	53.25 ± 14.01
Age(death)	21	54.55 ± 11.49
Tumor size(diameter/cm)	91	3.63 ± 2.43
Follow-up(month)	62	26.52 ± 46.84
Postoperative metastasis time(month)	24	18.72 ± 15.04

### Tumor characteristics

**Fig. 5:**
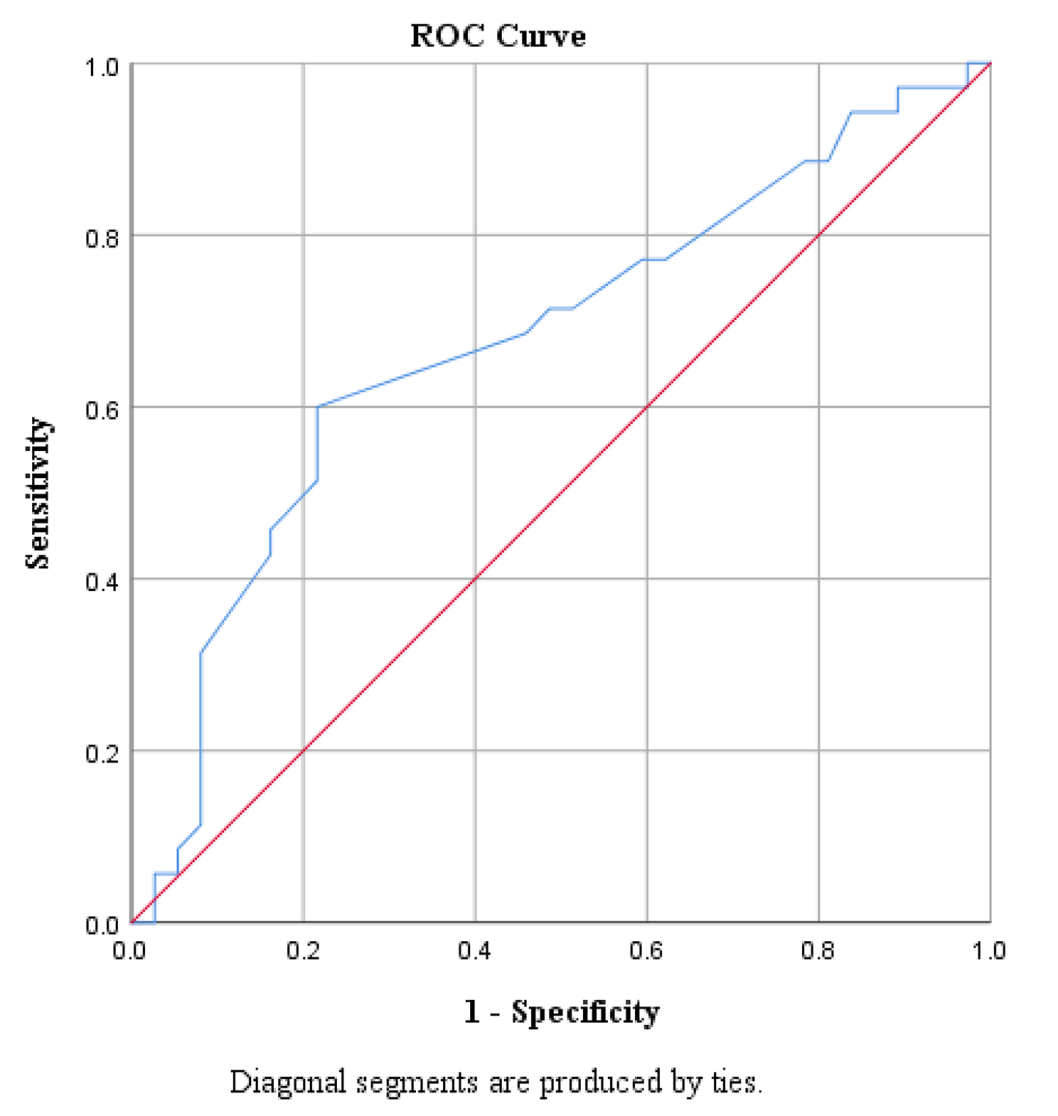
ROC Curve

In 91 samples detailing the tumor, the average tumor diameter was 3.63 ± 2.43 cm (Table 1). Tumors tended to occur on the right side in 53(55.21%) cases and on the left side in 41(42.71%) cases. Of the samples that clearly provided the onset quadrant, 39(53.42%) cases occurred in the outer upper quadrant, 7(9.59%) cases in the inner upper quadrant, 1(1.37%) case in the areolar region, 7(9.59%) cases in the outer lower quadrant, 2(2.74%) cases in the inner lower quadrant, 18(24.66%) cases involving multiple quadrants, and 2(2.74%) cases in the accessory breast (Tables 2 and 3). With that exception of 16 patient who were unclear whether axillary lymph node biopsy or dissection had been performed, of the 82 patients, 42(51.22%) had lymph node metastasis, 2(2.44%) had multiple systemic metastases, and 38(46.34%) had no metastasis (Table 4). We analyzed the ROC curve of the 72 samples that provided the maximum tumor diameter and metastasis results (Fig. 5). By calculating the maximum Jordan index (0.384), we found that when the tumor diameter>3.25 cm, it was easy to metastasize. The ROC curve area (0.676) < 0.7 indicated the general degree of differentiation (P < 0.05; P = 0.01), indicating statistical significance.

**Table 2:** Site of onset

Site of onset	No.of patients	Percentage
Left breast	41	42.71%
Right breast	53	55.21%
Left accessory breast	1	1.04%
Right accessory breast	1	1.04%

**Table 3:** Onset quadrant

Quadrant	No.of patients	Percentage
Outside up	39	53.42%
Outside down	7	9.59%
Inside	7	9.59%
Inner bottom	2	2.74%
Central area	2	2.74%
Areola region	1	1.37%
Multiple quadrants	18	24.66%
Accessory breast	2	2.74%

### Pathology

All the patients included in the study were diagnosed with LRCB. According to the molecular classification standard of breast cancer, among the 46 patients who clearly provided the expression conditions of Her-2 and HR, Her-2(+) and HR (-) 17 cases (36.96%) were tri-negative breast cancer 11 cases (23.91%) were luminal A breast cancer 8 cases (17.39%) were luminal B breast cancer 10 cases (21.74%) (Table 4). Of the 46 patients that provided information on Her-2 expression, 26(56.52%) were positive for molecular expression; Thirty-five of 54 patients (64.81%) who provided ER expression were negative; Of the 56 patients who provided PR expression, 38(67.86%) were negative (Table 4), 14(93.33%) of the 15 patients who provided samples for P53 expression were positive, and 4(100%)of the 4 patients who expressed Topo II were all positive (Table 5). Among the pathological-specific stains of LRCB, 36(85.71%) of 42 patients with fat staining were positive, and 40(86.96%) of 46 patients with PAS staining were negative (Table 4).

**Table 4:** Pathology

Project		No.of patients	Total	Percentage
Sort				
Typing	Her-2(+) ,HR(+)	8	46	17.39%
	Her-2(+) ,HR(−)	17	46	36.96%
	Basal-Like Breast Cancer	11	46	23.91%
	Luminal A Breast Cancer	8	46	17.39%
	Luminal B Breast Cancer	10	46	21.74%
Axillary Lymph Node Metastasis	Yes	42	82	51.22%
Fat staining positive	Yes	36	42	85.71%
PAS Negative	Yes	40	46	86.96%

**Table 5:** Molecular Typing

Project	No.of patients	Total	Percentage
Her-2 Positive	26	46	56.52%
ER Negtive	35	54	64.81%
PR Negtive	38	56	67.86%
P53 Positive	14	15	93.33%
TopoII Positive	4	4	100.00%

### Treatment

Of the 88 patients who provided a detailed surgical protocol, 41(46.59%) underwent modified radical mastectomy, and 21(23.86%) underwent radical mastectomy. The choice of surgical approach was not only closely related to the patient’s condition but also to the era at that time. Of the 41 patients who received postoperative treatment, 22(53.66%) received chemotherapy + local radiotherapy postoperatively, 15 (34.15%) received chemotherapy only, 3(7.32%) received local radiotherapy only, 1(2.44%) received neoadjuvant chemotherapy, no chemotherapy was administered postoperatively, and 1(2.44%) received no postoperative treatment. However, among the 98 cases included, there were 69 cases (70.41%) in China and 78.57% in Asia (Fig. 4). The summary of treatment methods may be incomplete or biased due to different countries.

### Prognosis

**Fig. 6:**
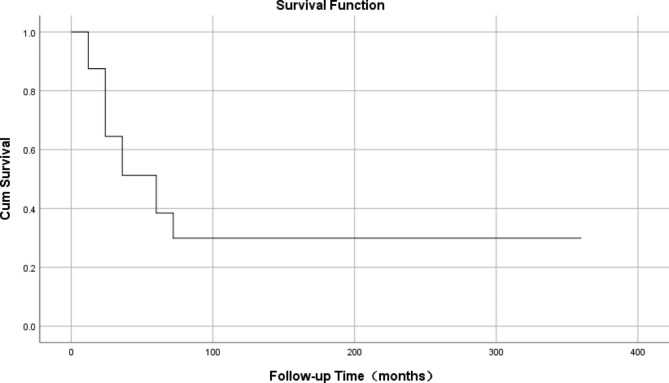
Median survival time

**Fig. 7:**
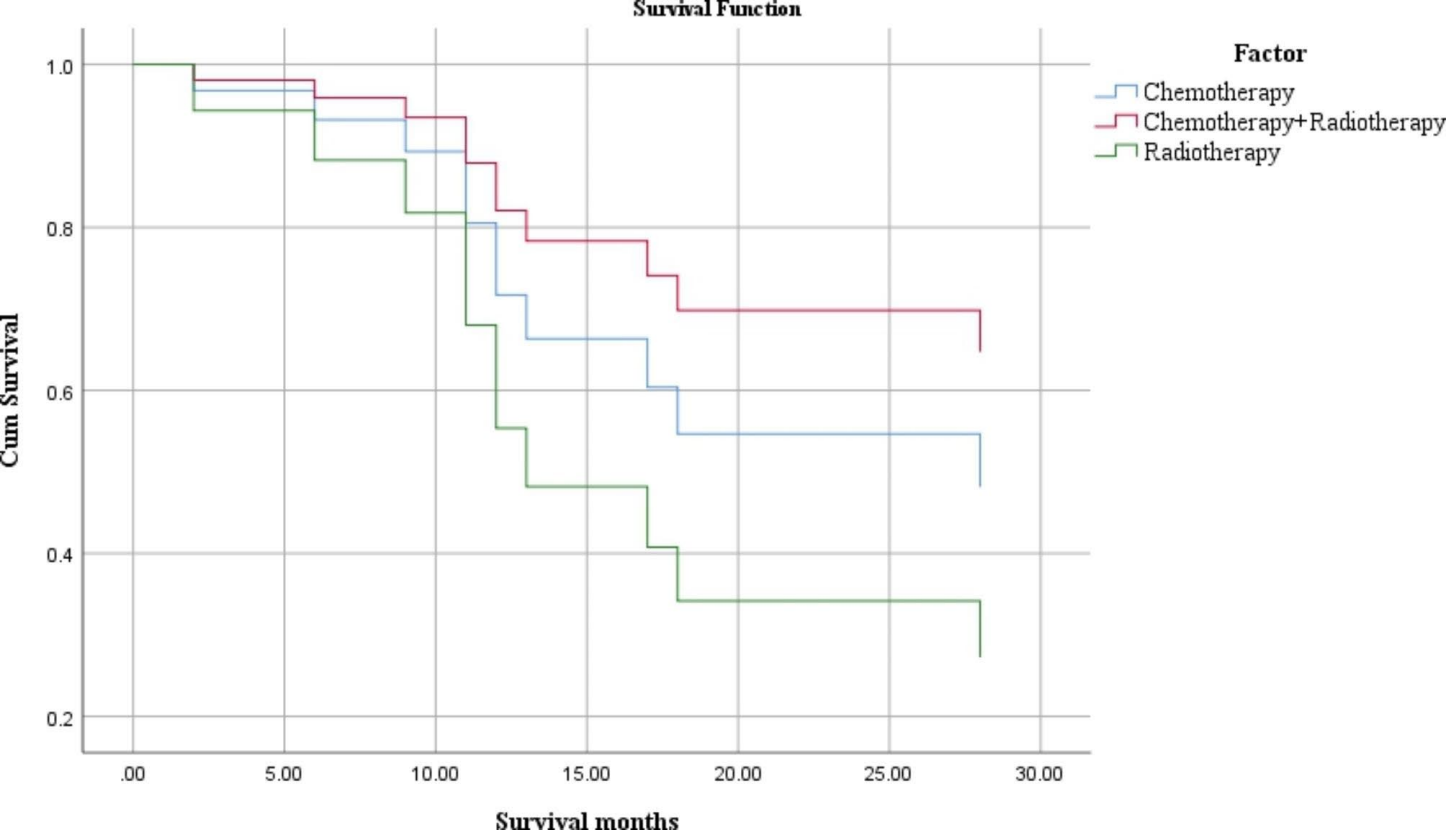
Chemotherapy、Chemotherapy and Radioiodine、Radioiodine AND Median survival time

Statistical analysis of the samples that clearly provided the follow-up time and outcome showed that the average follow-up time was 26.52 ± 46.84 months (Table 1), the median survival time was 49.17 months (Fig. 6), the shortest survival time was 3 weeks and the longest survival time was 20 years. Among 64 patients with definite follow-up results, 19(29.69%) died, ranging from 0.75 to 48 months after surgery, with an average of 13.96 months after operation. Most deaths occur in patients with distant metastases. Of the 18 patients with distant metastases, eight (44.44%) died. The most common site of metastasis was the lungs, followed by the liver. Of the eight cancer-type deaths diagnosed, five (62.50%) were Her-2(+) and HR (-), two (25%) were tri-negative, one (12.50%) was luminal A, and there were no luminal B patients. Among the treatment options for postoperative patients, the mortality rate (26.67%) for patients who received chemotherapy plus radiotherapy simultaneously was the lowest (Table 6). According to the analysis of survival results, the survival time after surgery with radiotherapy and chemotherapy was the longest (Table 7; Fig. 7). However, the relationship between metastasis, treatment plan, molecular typing, and death was not statistically significant, which may be due to the small sample size.

**Table 6:** Relationship between influencing factors and death

Influencing factors		No.of patients	Total	Percentage
Sort				
Metastasis	Yes	8	18	44.44%*
Chemotherapy and Radioiodine	Yes	4	15	26.67%*
Chemotherapy	Yes	6	22	27.27%*
Radioiodine	Yes	2	3	66.67%*
Typing	Her-2(+) ,HR(−)	5	8	62.50%
	Basal-Like Breast Cancer	2	8	25.00%
	Luminal A Breast Cancer	1	8	12.50%
	Luminal B Breast Cancer	0	16	0

*The difference was not statistically significant

**Table 7:** Life Table

		Interval Start Time	Number Entering Interval	Number Exposed to Risk			
	Chemotherapy	0	19	16.5			
First-order Controls		12	10	8	Number of Terminal Events	Cumulative Proportion Surviving at End of Interval	
Factor		24	4	3	4	0.76	
		36	2	1.5	2	0.57	
		48	1	0.5	0	0.57	
	Chemotherapy and Radioiodine	0	12	10	0	0.57	
		12	8	7.5	0	0.57	
		24	5	4.5	0	1	
		36	3	2.5	2	0.73	
		48	2	2	1	0.57	
		60	2	1.5	0	0.57	
		72	1	1	0	0.57	
		84	1	1	0	0.57	
		96	1	0.5	0	0.57	
	Radioiodine	0	3	3	0	0.57	
		12	2	2	0	0.57	
		24	1	0.5	1	0.67	
					1	0.33	
					0	0.33	

Among the patients who received chemotherapy and/or radiotherapy after surgery, two (66.67%) of the three patients who only received radiotherapy had distant metastasis. Three (20%) of the 15 patients who received chemotherapy and radiotherapy simultaneously had metastases. Of the 22 patients who received chemotherapy only, three (9.09%) had metastasis. Among the patients who clearly provided the pathological type and reported metastasis, Her-2(+) and HR (-) breast cancer patients had the most metastasis, accounting for 66.67%. This was followed by luminal B and tri-negative subtypes, accounting for 16.67% (Table 8). However, the relationship between molecular typing, treatment plan, and metastasis was not statistically significant, which may be related to the small sample size.

**Table 8:** Relationship between influencing factors and Metastasis

		No.of patients	Total	Percentage(%)
Influencing factors				
Chemotherapy and Radioiodine	Yes	3	15	20%*
Chemotherapy	Yes	3	33	9.09%*
Radioiodine	Yes	2	3	66.67%*
Typing	Her-2(+) ,HR(−)	4	6	66.67%
	Basal-Like Breast Cancer	1	6	16.67%
	Luminal A Breast Cancer	1	6	16.67%
	Luminal B Breast Cancer	0	12	0

*The difference was not statistically significant

## Discussion

Regarding the naming of LRCB, some researchers believe that lipid-secreting breast cancer is more appropriate. The appearance of lipids in breast cancer cells is the secretion product of the epithelial cells of the terminal duct, which is similar to the secretion activity of the mammary epithelium in the third trimester and lactation, but not steatosis. An ultrastructural study further confirmed this conjecture [3]. Ramos [2] believed that the needle-like crystalline substance in mitochondria is a type of hydroxyapatite and confirmed by electron microscopy that the lipid in LRCB cells was a secretion, not degeneration, because secretory vacuoles containing fat and mucin, lack of autophagy vacuoles, and the existence of rough endoplasmic reticulum were observed near the Golgi apparatus. Wrba et al. [4]reported that, in their cases, no immunohistochemical or ultrastructural findings could support lipid secretion by cancer cells. Therefore, “lipid-rich cancers " are considered a more reasonable description of this type of breast cancer than “lipid-secreting cancers “. The term “lipid-rich” is now preferred because it only describes the existence of lipid substances in cells and not active lipid secretion, unless there is evidence of active lipid secretion in tumor cells [5]. Nevertheless, the definition of LRCB remains controversial because the percentage of vacuole cells and source types of lipid vacuoles are still unclear [6].

Breast mass was the main symptom at the onset of LRCB. Most patients complain of a breast mass. Few patients with metastases visited their doctors with the main complaint of tumor metastasis symptoms.

LRCB is more common in unilateral breast masses, with or without pain. Color Doppler ultrasonography of the breast is the first choice for patients.

The literature review shows that the age of onset of LRCB ranges from 10 to 83 years, and the peak age of onset is 40 to 55 years. Most LRCB patients are women, but men are even rarer. To date, only two male patients have been reported. The main clinical manifestation of LRCB is a breast mass, which can be involved in both breasts successively, most of which are unilateral. Occasionally, the right breast occupies space at the same time as the left breast, which is more common in the outer upper quadrant, followed by multi-quadrant occupying space. The diameter of the masses ranged from 0 cm to 12–3 cm, with 2–3 cm being the most common. LRCB usually has a short course of disease, usually within six months, and lymphatic or blood metastasis can occur in the early stage of LRCB. Lymph node metastasis occurs mostly in the axillary or supraclavicular region, and blood metastasis occurs mostly in the lung and liver. LRCB is characterized by high malignancy, rapid development, easy metastasis, and a poor prognosis.

Because the number of lipid-containing cells needed to confirm the diagnosis of lipid-rich breast cancer is still unclear, the definition of a tumor remains unclear [6]. However, studies have shown that more than 90% of tumor cells should contain lipid droplets to be considered lipid-rich cancer [7]. The source of lipids in tumor cells is also unclear, which is one of the reasons why there is no clear standard for diagnosis. The cell types of LRCB can be divided into the following three types: histiocytic, sebaceous gland-like, and apocrine gland [8], and two or three types usually exist simultaneously. The pathological features of the tumor are hard texture, light yellow sections, unclear boundaries with surrounding tissues, and greasiness. Under a light microscope, a small amount of fibrous tissue was observed, and the tumor cells were arranged in irregular nests. Normal breast epithelial cells not only have the ability to synthesize proteins and sugars but also synthesize lipids. Some breast cancer cells synthesize and secrete these substances. Gland or small papilla with obvious separation between the main and stroma, showing infiltration and diffuse growth. The cancer cells are large, round or oval, with clear cell membranes, irregular nuclei, mild to moderate heteromorphism, prominent nucleoli, abundant and transparent cytoplasm, foamy or honeycomb, lightly stained or granular, and the cells are arranged in glandular, banded, or nested shapes, with remarkable secretion results. Fat staining was strongly positive, but PAS staining was negative. Studies have shown that there are many lipid droplets of different sizes near the Golgi apparatus, and there are many electron-dense substances in the smooth endoplasmic reticulum, which indicates that a large amount of lipid is the product secreted by cancer cells and not caused by degeneration [9].

The differential diagnosis of LRCB includes primary and secondary vacuolar or clear-cell breast tumors as well as glycogen-rich carcinoma, apocrine carcinoma, and secretory carcinoma, all of which have different metabolites in the foamy cytoplasm [10]. ① Secretory carcinoma: The cells of LRCB are similar to those of secretory carcinoma in that the cytoplasm of the cancer cells is empty and light, the arrangement is an acinar structure, and PAS staining is negative. However, secretory carcinomas generally exhibit clear boundaries, good activity, and slow growth. ② Fat necrosis of the breast: Most of the lipid-phagocytic cells after fat necrosis are foamy, but there are no types. There were obvious inflammatory and foreign giant cells in the lesions. ③ Great Khan adenocarcinoma: PAS staining of great Khan adenocarcinoma was positive, and GCDFP-15 was mostly positive on immunohistochemistry [11]. ④ Glycogen-rich carcinoma of the breast: The cytoplasm of this tumor cell is transparent, with positive PAS staining, which can be digested by amylase, but with negative fat staining, so that it can be distinguished from LRCB [12].

Topoi-somerase II (Topo II) is a DNA topoisomerase encoded by Topo II, which has obvious cell cycle specificity. As a target of anthracyclines, related research has attracted increasing attention and has been used as a predictive marker in research and clinical practice. However, Topo II-α has not been widely detected in LRCB. Because it is adjacent to the Topo II-α gene, the probability of amplification of Topo II-α gene is 24.3% ~ 55% in primary breast cancer amplified by the HER2 gene [13]. The amplification of the HER-2 gene is highly correlated with the expression of Topo II protein and RNA levels, suggesting that the amplification of HER-2 gene may stimulate the expression of Topo II protein [14]. By analyzing the expression of HER-2 and Topo II-α in breast cancer, we found that the overexpression of HER-2 and amplification of TopoII-α were related to the poor prognosis of LRCB, and the sensitivity of these patients to anthracycline-containing drug regimens increased [15, 16].

P53 is one of the tumor suppressor genes with the highest correlation with human tumors discovered to date. The higher the expression of P53, the worse the differentiation of tumor tissues, the higher the malignancy, and the worse the prognosis. Among them, 50% of tumors have a P53 mutation [17]. Mutant P53 expression suggests that patients are more sensitive to anthracyclines [18]. The results showed that the expression of Topo II was related to the expression of P53, suggesting that patients with co-expression of Topo II and P53 may benefit more from anthracyclines [19]. Topo II was significantly positively correlated with P53 expression, and the co-expression rate was significantly higher than the non-co-expression rate (P < 0.05) [20]. We can think that when Topo ii is expressed, there is a synergistic relationship with P53, and the probability of co-expression of both of them may increase. P53 may help Topo ii predict the efficacy of anthracyclines.

The expression of TOPO II in breast cancer can predict the biological behavior of breast cancer, especially anthracycline chemotherapy drugs, but the single index is insufficient for predicting the curative effect [21]. Meta-analysis of the correlation between P53 expression in breast cancer tissues and the efficacy of neoadjuvant chemotherapy also shows that P53 can be used as an indicator of the sensitivity of neoadjuvant chemotherapy for breast cancer [22]. At present, the molecular biological detection factors ER, PR and HER-2, which have been widely used, have important guiding significance for the prognosis and treatment of breast cancer. However, combined detection of multiple indicators can more accurately evaluate the curative effect and prognosis, thus helping to formulate a treatment plan, especially for HER-2 overexpressed breast cancer.

At present, LRCB lacks unified treatment plan and standard, but we generally consider that once the diagnosis is confirmed, modified radical mastectomy or radical mastectomy with chemotherapy and/or radiotherapy should be performed. Because of the high rate of axillary lymph node metastasis in LRCB, axillary lymph node dissection is usually necessary. Lipid-rich cancers can metastasize immediately after surgery. The most common metastatic sites include the lungs, liver, and bones. Therefore, systemic therapy plays an important role in treatment. Lipid-rich cancers are usually positive for HER-2, but negative for hormone receptors, which is consistent with the results of this study. Because hormone receptors are negative, the effects of endocrine therapy are often limited. Therefore, chemotherapy is the most important component of LRCB. Compared with doxorubicin, lipid-rich carcinoma is more sensitive to platinum and molecular-targeted drugs (such as paclitaxel and vincristine) [5]. In addition, the positive rate of Topo II in the LRCB was high. Therefore, paclitaxel combined with anthracycline is currently recommended as chemotherapy. For patients with HER-2 positive or strongly positive expression, trastuzumab is now advocated for gene-targeted therapy. According to the results of this study, the metastasis rate of patients who only received chemotherapy was the lowest, but considering that the sample size of this study was small and the prognosis of many patients was not detailed, the results of this study are for reference only. Considering the high degree of malignancy and poor prognosis of LRCB, according to the guidelines for the treatment of breast cancer, patients with positive axillary lymph nodes should be treated with radiotherapy.

The prognosis of LRCB is poor and there is currently no targeted treatment. Once the diagnosis is made, measures should be taken according to the principle of high-grade breast cancer treatment, and patients with surgical indications should undergo radical mastectomy. LRCB surgery often results in local recurrence. The first metastatic site is usually the axillary lymph node, which easily metastasizes to the orbital soft tissue. It can also metastasize to the contralateral breast, lung, liver, and bone along the blood channel, and half of these patients die within two years. Therefore, great importance should be attached to this disease. Although only a few cases of LRCB have been reported, the prognosis of this cancer is very poor. The overall 2-year and 5-year survival rates were 64.6% and 33.2%, respectively [10], and the median survival time was 49.17 months. Our research found that 52.05% of patients had axillary lymph node metastasis, and most of the postoperative metastasis occurred at 32.13 ± 17.44 months. LRCB tends to express HER-2 at a high level, which is also the reason for its poor prognosis and short disease-free survival [23].

## Summary

In summary, this study analyzed the clinical features, pathology, treatment, and prognosis of LRCB and emphasized that LRCB is a rare type of breast cancer. Owing to its poor prognosis, a correct diagnosis is necessary for this extremely rare tumor. Early diagnosis and timely treatment may help improve the overall survival and disease-free survival of patients with LRCB.

**Table 9:** Origin of 98 cases of LRCB

Number	Article	First author	Magazine	Publication Time
1	A case report of lipid-rich carcinoma of the breast including histological characteristics and intrinsic subtype profile	Kimura, A.	Case Reports in Oncology	2011
2	Clear cell carcinoma of breast lipid-rich variant	Kini, H.	J Cancer Res Ther	2019
3	Ductal carcinoma of male breast with prominent lipid-rich component	Mazzella, F. M.	BMC Cancer	1995
4	Fine-Needle Aspiration of an Apocrine Breast Carcinoma With Multivacuolated, Lipid-Rich, Giant Cells	Maire A	Diagn Cytopathol	1988
5	Her-2 neu negative lipid rich breast carcinoma in an immunocompromised patient	Sirohi, D.	Human Pathology: Case Reports	2015
6	Intraductal Lipid-Rich Carcinoma of the Breast with a Component of Glycogen-Rich Carcinoma	Yoshitaka Kurisu	J Breast Cancer	2012
7	Lipid secreting breast carcinoma in childhood: A case report	Balik, E.	European Journal of Pediatric Surgery	1993
8	Lipid-Rich Carcinoma of Breast: A Case Report With Fine Needle Aspiration Cytology	Catalina-Fernández, I.	Diagnostic Cytopathology	2009
9	Lipid-rich carcinoma of male breast in Chinese: a case report and literature review	shujian xu	Int J Clin Exp Med	2015
10	Lipid-rich Carcinoma of the Breast : a Case Report	P. Shi	Acta chir belg	2008
11	Lipid-rich carcinoma of the breast that is strongly positive for estrogen receptor: a case report and literature review	Takaaki Oba	Onco Targets Ther	2016
12	Lipid-Rich Carcinoma of the Breast With Unusual Clinical and Histopathological Features	Balan Louis Gaspar, MD	International Journal of Surgical Pathology	2016
13	Lipid-rich carcinoma of the breast: A report of two cases and a literature review	yizi cong	Oncol Lett	2015
14	Lipid-rich carcinoma of the breast: A report of two cases and a literature review	yizi cong	Oncol Lett	2015
15	Lipid-Rich versus Lipid-Secreting Carcinoma of the Mammary Gland	F. Vera-Sempere	Path. Res. Pract	1985
16	Lipid-Secreting Carcinoma of the Breast: A Case Report and Review of the Literature	Yoshihisa Umekita	Breast Cancer	1998
17	Metaplastic lipid-rich carcinoma of the breast	Zsuzsanna Varga	Pathology International	1998
18	Pathologic quiz case: a 62-year-old woman with a 4.5-cm nodule in the right breast. Lipid-rich breast carcinoma	Reis-Filho, J. S.	Arch Pathol Lab Med	2003
19	The problematic interpretation of foamy cells in breast fine needle aspirates. A report of two cases	Gottschalk-Sabag, S.	Acta Cytol	1997
20	Unusual occurrence of rare lipid-rich carcinoma and conventional invasive ductal carcinoma in the one breast: case report	KatarinaMachalekova	Case Rep Pathol.	2012
21	A non-invasive form of lipid-secreting carcinoma of the breast	Yoshika Nagata	Breast Cancer	2012
22	Lipid-secreting mammary carcinoma. Report of a case associated with Paget’s disease of the nipple	M H ABOUMRAD	Cancer	1963
23	Lipid-rich histology in a basal-type immuno-profile breast carcinoma: a clinicopathological histochemical and immunohistochemical analysis of a case	Serena Russo	Rare Tumors	2009
24	Ultrastructural and immunohistochemical characteristics of lipid-rich carcinoma of the breast. Virchows Arch A Pathol Anat Histopathol	Friedrich Wrba	Virchows Archiv A Pathol Anat	1988
25	Ultrastructural and immunohistochemical characteristics of lipid-rich carcinoma of the breast. Virchows Arch A Pathol Anat Histopathol	Friedrich Wrba	Virchows Archiv A Pathol Anat	1988
26	Ultrastructural and immunohistochemical characteristics of lipid-rich carcinoma of the breast. Virchows Arch A Pathol Anat Histopathol	Friedrich Wrba	Virchows Archiv A Pathol Anat	1988
27	Ultrastructural and immunohistochemical characteristics of lipid-rich carcinoma of the breast. Virchows Arch A Pathol Anat Histopathol	Friedrich Wrba	Virchows Archiv A Pathol Anat	1988
28	Ultrastructural and immunohistochemical characteristics of lipid-rich carcinoma of the breast. Virchows Arch A Pathol Anat Histopathol	Friedrich Wrba	Virchows Archiv A Pathol Anat	1988
29	Lipid-rich versus lipid-secreting carcinoma of the mammary gland	Vera-Sempere F	Pathol Res Pract.	1985
30	Fine needle aspiration cytology of lipid-secreting carcinoma of the breast	Aida Y	Acta Cytol	1993
31	Fine needle aspiration cytology of lipid-secreting breast carcinoma. A case report	Insabato L	Acta Cytol	1993
32	Unusual variant of lipid-rich mammary carcinoma	Lim-Co	Arch Pathol Lab Med	1978
33	A unique case of breast carcinoma producing pancreatic-type iso-amylase	WEITZEL J. N	GASTROENTEROLOGY	1988
34	Secreting Lipid Breast Cancer	Ren zhi	Shanxi Medicine	1990
35	A case of lipid-rich carcinoma of accessory breast	Wu Jundong	Chinese Journal of General Surgery.	2004
36	Secretory lipid carcinoma of breast: a case report	Lin Lizhu	China tumor clinic	2000
37	Secretory lipid carcinoma of breast: a case report	Shen weijiang	Chinese journal of clinical and experimental pathology	1999
38	Secretory lipid carcinoma of breast: a case report	Wang Liming.	Clinical study of China tumor	2006
39	Secretory lipid carcinoma of breast: a case report	Cui Xiuzhen	Journal of Binzhou Medical College	1988
40	Diagnosis and treatment of 5 cases of secretory lipid carcinoma of breast	Wu yigang	World Journal of Integrated Traditional Chinese and Western Medicine	2009
41	Diagnosis and treatment of 5 cases of secretory lipid carcinoma of breast	Wu yigang	World Journal of Integrated Traditional Chinese and Western Medicine	2009
42	Diagnosis and treatment of 5 cases of secretory lipid carcinoma of breast	Wu yigang	World Journal of Integrated Traditional Chinese and Western Medicine	2009
43	Diagnosis and treatment of 5 cases of secretory lipid carcinoma of breast	Wu yigang	World Journal of Integrated Traditional Chinese and Western Medicine	2009
44	Diagnosis and treatment of 5 cases of secretory lipid carcinoma of breast	Wu yigang	World Journal of Integrated Traditional Chinese and Western Medicine	2009
45	Light and electron microscopic observation of lipid-secreting carcinoma of breast: a case report	Wu Lili	Academic journal of second military medical university	1997
46	Clinicopathological analysis of lipid-secreting carcinoma of breast	Huai Jianguo	Clinicopathological analysis of lipid-secreting carcinoma of breast	2009
47	Clinicopathological analysis of lipid-secreting carcinoma of breast	Huai Jianguo	Clinicopathological analysis of lipid-secreting carcinoma of breast	2009
48	Clinicopathological analysis of lipid-secreting carcinoma of breast	Huai Jianguo	Clinicopathological analysis of lipid-secreting carcinoma of breast	2009
49	A Case of Secretory Lipid Carcinoma of Breast	Shixuehai	Jilin medicine	1986
50	Lipid-rich breast cancer: a case report	Fu yishan	Journal of chongqing medical university	2006
51	Lipid-rich breast cancer: a case report	Liu huijie	Chinese clinical oncology	2012
52	Lipid-rich breast cancer: a case report	Xu youkun	Clinical military doctor	2003
53	Lipid-rich breast cancer: a case report	Li jinyun	Yunnan medicine	2000
54	Lipid-rich breast cancer: a case report	Sun wei	Chinese journal of coal industry medicine	2005
55	Lipid-rich carcinoma of breast: a case report	Wang shengli	Journal of baotou medical college	2017
56	Lipid-rich carcinoma of breast: a case report and literature review	Dong weilu	Tumor imaging	2020
57	Lipid-rich breast cancer: a case report	Zhou guitai	Journal of Xianning University (Medical Edition)	2010
58	Lipid-rich breast cancer: a case report	Li tongchang	Journal of Hebei Staff Medical College	1995
59	Lipid-rich breast cancer: a case report	Li shuguang	China tumor clinic	2003
60	Lipid-rich breast cancer: a case report	Liu liping	Journal of clinical ultrasound	2008
61	Lipid-rich carcinoma of breast: a case report and literature review	He li	Chongqing medicine	2021
62	Lipid-rich carcinoma of breast: a case report and literature review	Jiang wei	Journal of modern oncology	2011
63	Lipid-rich carcinoma of breast: a case report	Chen jumin	Basic and Clinical Practice of General Surgery in China	2006
64	Lipid-rich carcinoma of breast: a case report	Li guanglian	Journal of Chinese physician	2005
65	Lipid-rich carcinoma of breast: a report of two cases	Gao liya	Cancer and Rehabilitation in China	1996
66	Lipid-rich carcinoma of breast: a report of two cases	Gao liya	Cancer and Rehabilitation in China	1996
67	A case of lipid-rich carcinoma of breast	Wei rui	Chinese journal of radiation oncology	2001
68	Lipid-rich carcinoma of breast (report of 7 cases)	Geng cuizhi	Journal of Hebei Medical College	1995
69	Lipid-rich carcinoma of breast (report of 7 cases)	Geng cuizhi	Journal of Hebei Medical College	1995
70	Lipid-rich carcinoma of breast (report of 7 cases)	Geng cuizhi	Journal of Hebei Medical College	1995
71	Lipid-rich carcinoma of breast (report of 7 cases)	Geng cuizhi	Journal of Hebei Medical College	1995
72	Lipid-rich carcinoma of breast (report of 7 cases)	Geng cuizhi	Journal of Hebei Medical College	1995
73	Lipid-rich carcinoma of breast (report of 7 cases)	Geng cuizhi	Journal of Hebei Medical College	1995
74	Lipid-rich carcinoma of breast (report of 7 cases)	Geng cuizhi	Journal of Hebei Medical College	1995
75	Lipid-rich breast cancer: a case report	Wang zhenyi	The practical journal of cancer	1993
76	Lipid-rich carcinoma of breast: a case report	Yu xishen	Chinese journal of clinical and experimental pathology	1987
77	A case of bilateral lipid-rich breast cancer	He huijun	Chinese journal of surgery	2007
78	Bilateral breast lipid carcinoma: a case report	Zhao rongxiu	Occupation and health	2003
79	A case of lipid-secreting breast cancer without mass	Qu lesong	National medical journal of china	2004
80	Lipid cell carcinoma of axilla accessory breast: a case report	zheng tiangui	Henan Journal of Oncology	2000
81	Lipid-rich carcinoma of right breast with repeated left breast cancer: a case report	Ming hui	Practical journal of medicine & pharmacy	2012
82	Lipid cell carcinoma of left breast: a case report	Liu yuesong	Jiangsu medical journal	1998
83	A Case of Giant Lipoid Carcinoma of Breast	Li yu’e	Chinese Journal of Breast Diseases (Electronic Edition)	2015
84	Lipid Secreting Breast Cancer (A Case Report)	Wang xianhu	The practical journal of cancer	1988
85	Lipid Secreting Breast Cancer (A Case Report)	Wang xianhu	The practical journal of cancer	1988
86	Lipid Secreting Breast Cancer (A Case Report)	Wang xianhu	The practical journal of cancer	1988
87	Lipid Secreting Breast Cancer (A Case Report))	Wang xianhu	The practical journal of cancer	1988
88	A case report of lipid-rich breast cancer	Guo lingxin	Chinese journal of pathology	1984
89	Lipid carcinoma of breast —— A case report	Sun yukai	Chinese journal of oncology	1984
90	Clinicopathological analysis of lipid-rich breast cancer	Wang hai	Chinese journal of cancer prevention and treatment	2009
91	Clinicopathological analysis of lipid-rich breast cancer	Wang hai	Chinese journal of cancer prevention and treatment	2009
92	Clinicopathological analysis of lipid-rich breast cancer	Wang hai	Chinese journal of cancer prevention and treatment	2009
93	Clinicopathological analysis of lipid-rich breast cancer	Wang hai	Chinese journal of cancer prevention and treatment	2009
94	Clinicopathological analysis of lipid-rich breast cancer	Wang hai	Chinese journal of cancer prevention and treatment	2009
95	Clinicopathological analysis of lipid-rich breast cancer	Wang hai	Chinese journal of cancer prevention and treatment	2009
96	Clinicopathological analysis of lipid-rich breast cancer	Wang hai	Chinese journal of cancer prevention and treatment	2009
97	Clinicopathological analysis of lipid-rich breast cancer	Wang hai	Chinese journal of cancer prevention and treatment	2009
98	A case of intraciliary metastasis of lipid-rich breast cancer	Liu ling	Chinese journal of ophthalmology	2001

## Data Availability

The data described in this article can be freely and openly accessed at the official website of PubMed, ClinicalTrials.gov, Embase, Cochrane Library, and China National Knowledge Infrastructure databases.

## Abbreviations

LRCB: Lipid-rich carcinoma of the breast
CNKI: China National Knowledge Infrastructure
SPSS: Statistical Product Service Solutions
